# Pathogenicity of Genetically Similar, H5N1 Highly Pathogenic Avian Influenza Virus Strains in Chicken and the Differences in Sensitivity among Different Chicken Breeds

**DOI:** 10.1371/journal.pone.0153649

**Published:** 2016-04-14

**Authors:** Aya Matsuu, Tomoko Kobayashi, Tuangthong Patchimasiri, Takashi Shiina, Shingo Suzuki, Kridsada Chaichoune, Parntep Ratanakorn, Yasuaki Hiromoto, Haruka Abe, Sujira Parchariyanon, Takehiko Saito

**Affiliations:** 1 Thailand-Japan Zoonotic Diseases Collaborating Center (ZDCC), Kasetlang, Chatuchak, Bangkok, Thailand; 2 Research Team for Zoonotic Diseases, National Institute of Animal Health, National Agriculture and Food Research Organization (NARO), Kannondai, Tsukuba, Ibaraki, Japan; 3 Transboundary Animal Diseases Research Center, Joint Faculty of Veterinary, Kagoshima University, Korimoto, Kagoshima, Japan; 4 Department of Molecular Life Science, Tokai University School of Medicine, Shimokasuya Isehara, Kanagawa, Japan; 5 The Monitoring and Surveillance Center for Zoonotic Diseases in Wildlife and Exotic Animals, Faculty of Veterinary Science, Mahidol University, Salaya, Phuttamonthon, Nakhon Pathom, Thailand; 6 National Institute of Animal Health, Kasetklang, Chatuchak, Bangkok, Thailand; 7 Influenza and Prion Disease Research Center, National Institute of Animal Health, National Agriculture and Food Research Organization (NARO), Kannnondai, Tsukuba, Ibaraki, Japan; University of Edinburgh, UNITED KINGDOM

## Abstract

Differences in the pathogenicity of genetically closely related H5N1 highly pathogenic avian influenza viruses (HPAIVs) were evaluated in White Leghorn chickens. These viruses varied in the clinical symptoms they induced, including lethality, virus shedding, and replication in host tissues. A comparison of the host responses in the lung, brain, and spleen suggested that the differences in viral replication efficiency were related to the host cytokine response at the early phase of infection, especially variations in the proinflammatory cytokine IL-6. Based on these findings, we inoculated the virus that showed the mildest pathogenicity among the five tested, A/pigeon/Thailand/VSMU-7-NPT/2004, into four breeds of Thai indigenous chicken, Phadu-Hung-Dang (PHD), Chee, Dang, and Luang-Hung-Khao (LHK), to explore effects of genetic background on host response. Among these breeds, Chee, Dang, and LHK showed significantly longer survival times than White Leghorns. Virus shedding from dead Thai indigenous chickens was significantly lower than that from White Leghorns. Although polymorphisms were observed in the *Mx* and MHC class I genes, there was no significant association between the polymorphisms in these loci and resistance to HPAIV.

## Introduction

The first highly pathogenic avian influenza (HPAI) H5N1 virus outbreak in poultry in Asia occurred at a goose farm in China in 1996 [1]. Serious H5N1 outbreaks in poultry accompanied by human casualties soon followed in Hong Kong in 1997 [2]. In 2002, outbreaks reemerged in both terrestrial poultry and wild waterfowl [3]. In late 2003, an outbreak of HPAI H5N1 in poultry was confirmed for the first time in South Korea. This was followed by eruptions in Vietnam, Japan, Thailand, Cambodia, China, Laos, and Indonesia at the beginning of 2004 [4]. By 2006, H5N1 HPAIV, the so-called Qinghai strain, had spread throughout Central and Southeast Asia, Europe, and Africa [5].

The H5N1 virus is endemic in poultry and has caused occasional infections in mammals. It poses a severe threat to the poultry industry in Southeast Asia and Africa. However, the mechanism(s) underlying the highly pathogenic manifestation of the H5N1 viruses in chickens has not been fully elucidated. Viruses like influenza are continually exposed to host immune systems, and their pathogenicity is thought to be related to viral replication, which either overwhelms the host immune system or causes it to malfunction. Various viral factors have been shown to be related to differences in pathogenicity. Specific amino acid substitutions in PB2 [6,7], PB1 [8,9], PB1-F2 [10], PA-X [11], NP [12], M1 [13], and NS1 [6,14] are correlated with viral replication or alterations in the host cytokine response against viral infection. Differences in HPAIV pathogenicity may also depend on the susceptibility of the host. Differences in the lethality of HPAIV infection among various lines or breeds of chicken have been described [15,16]. The host innate and adaptive immune responses to pathogens are related to the genetic background of the host. Mx protein, which is induced by type I interferon and is encoded by the *Mx1* gene [17], was reported to display intrinsic antiviral activity, and is associated with resistance to influenza virus infection in mammals [18]. *In vitro* studies have demonstrated that a substitution in the Mx protein (S631N) is associated with resistance to avian influenza virus infection in chickens [19,20]. However, some *in vivo* and *in vitro* studies have failed to demonstrate this association [15,21,22]. Major histocompatibility complex (MHC) haplotype is another candidate host resistant factor. In chicken, virtually all cells express MHC class I molecules, and they are thought to play a central role in MHC-restricted antigen presentation to cytotoxic T cells [23,24]. Studies suggest that chicken MHC haplotypes are associated with resistance or susceptibility to infectious diseases [24,25]. In a previous study, some Thai indigenous chickens were reported to have survived the H5N1 outbreaks during 2004–2005, and the B21 haplotype was suggested to be associated with the resistance to H5N1 infection [16]. However, no studies have been conducted to evaluate the differences in susceptibility among the various breeds of Thai indigenous chickens. The identification of a genetic factor affecting the resistance/susceptibility to HPAIV would aid in the elucidation of the mechanism underlying pathogenesis.

As the pathogenesis of HPAIV is complicated and is dependent on various host and viral factors, in this study, we compared the pathogenicity of five genetically related H5N1 (clade 1.1) viruses isolated from wild birds in Thailand between 2004 and 2005 to that in a commercial chicken breed, White Leghorn. Then, Thai indigenous chickens were inoculated with an H5N1 virus, and their sensitivity to infection and the genetic components related to the host response were examined.

## Materials and Methods

### Ethics Statement

All challenge experiments with H5N1 HPAIV were performed under the guidelines of the Animal Care and Use Committee. Our experimental protocol, describing that animals could die of the HPAIV infection and that animals would be euthanized if they manifested severe symptoms, such as inactivity, loss of appetite, loss of 20% or more body weight, etc., for 24 h, was reviewed and approved by the Faculty of Veterinary Science Animal Care and Use Committee. All experiments with live HPAIVs were performed in a biosafety level 3 containment laboratory at Mahidol University, Thailand, after approval by the faculty.

### Virus strains

The influenza viruses A/Openbill stork/Thailand/VSMU-16-AYA/2004 (openbill-1604), A/pigeon/Thailand/VSMU-7-NPT/2004 (pigeon-04), A/gray-crowed crane/Thailand/VSMU-4-CBI/2005 (crane-05), A/Openbill stork/Thailand/VSMU-6-BKK/2004 (openbill-6-04), and A/Silver Pheasant/Thailand/VSMU-1-CBI/2005 (pheasant-05) used in this study were isolated from wild birds through surveillance conducted during the HPAI outbreaks in poultry in Thailand from 2004 to 2005. The HA sequence of pigeon-04 was classified as clade 1 (WHO/OIE/FAO/H5N1 Evolution Working Group 2007) [26]. Virus stocks were propagated in Madin-Darby canine kidney (MDCK) cells, and the culture supernatant was harvested and stored at -80°C. The viral infectivity of each strain was determined by serial titration in 10–11-day-old embryonated eggs, and was expressed as 50% of the egg infective dose (EID_50_)/mL using the method reported by Reed and Muench [27]. The complete coding sequences of the gene segments were determined, and phylogenetic analyses were performed using the neighbor-joining method in MEGA5 (Molecular Evolutionary Genetics Analysis) [28].

### Experimental infection

Specific pathogen free (SPF) eggs of White Leghorn were obtained from BioLasco (Bangkok, Thailand) and were hatched in an incubator at the Faculty of Veterinary Medical Science, Mahidol University. At 3 weeks, the chickens were confirmed to be negative for H5-specific hemagglutination inhibition (HI) antibodies. The survival rate of chicken infected with HPAIV was investigated in 11 groups (n = 8) of 4-week-old White Leghorn chickens. All chickens were housed in isolators (CH8ISOL/CM12ISOL; Allentown Inc., Allentown, NJ, USA). The ventilation was set to negative pressure with high-efficiency particulate air (HEPA)-filtered air. The chickens were inoculated intranasally with 10^4^ or 10^6^ EID_50_/0.1 mL of HPAIV, and the control chickens were inoculated with 0.1 mL of medium. Blood samples were collected from all chickens prior to infection. Tracheal and cloacal swab samples were collected from the dead chickens immediately on the day of death for viral titration. Blood samples were collected from the surviving chickens on day 10. Sera were collected from all chickens before inoculation and from surviving and control chickens on day 10.

Viral dissemination and the host response were investigated in chickens infected with openbill-1604, pigeon-04, and crane-05, which demonstrated different pathogenicity characteristics. Four groups of three White Leghorn chickens were inoculated with HPAIV or were mock infected with medium, as described above. The chickens were euthanized with pentobarbital 24 h after inoculation, and tissue samples were obtained from the brain, lung, trachea, liver, spleen, and colon. These tissues samples were homogenized, and a 10% cell suspension was prepared in Modified Eagle’s Medium containing antibiotics for viral titration. Portions of the lung, spleen, and brain tissue samples were preserved in RNAlater solution (AM7021; Ambion, Austin, TX, USA) and stored at -80°C until RNA extraction.

One-day-old Chee, Dang, Phadu-Hang-Dum (PHD), and Luang-Hang-Khao (LHK) chickens were provided by the Department of Livestock Development (DLD), Thailand. At 14-weeks of age, the chickens were inoculated intranasally with 10^5^ EID_50_/0.1 mL of HPAIV, and the control group was inoculated with 0.1 mL of medium. The number of inoculated chickens per group was 13–16. Blood samples were collected for the HI test and were genotyping before inoculation. On day 10, tracheal and cloacal swab samples were collected from the dead and surviving chickens for virus titration, and blood samples were collected from surviving chickens.

All the chickens were monitored daily for clinical signs and survival until 10 days post-infection. The main expected clinical signs were face edema, swelling in the leg or face, and discoloration of the comb or leg cyanosis, weakness, anorexia, depression, ruffled feathers, twitch or convulsion, diarrhea, and death. A humane endpoint was applied when severe clinical signs were observed for more than 24 h, and they were euthanized by intraperitoneal injection of pentobarbital. The chickens that survived until 10 days after infection were also euthanized by pentobarbital injection (30–40 mg/kg).

#### Viral titration

The swab and homogenized tissue samples were stored at -80°C until titration into SPF eggs. The values for titration were calculated as described by Reed and Muench [27] and were expressed as EID_50_/mL and EID_50_/g, respectively.

#### Serological analysis

Sera were collected from all chickens before inoculation and from virus-infected and mock-inoculated chickens at 10 days post infection, and the sera were treated with receptor destroying enzyme to remove any nonspecific inhibitors. The HI test was performed using treated sera, chicken red blood cells, and four hemagglutination units of H5N1 HPAIV. Pre-inoculation sera from all chickens used in this study were determined to be serologically negative for H5-specific HI antibodies.

#### Evaluation of cytokine responses using quantitative real-time polymerase chain reaction (PCR) analysis

Total RNA was extracted from stored tissue with RNAlater using a commercial kit (RNeasy fibrous tissue mini kit; Qiagen, Hilden, Germany) according to the manufacturer’s instructions and was reverse transcribed to cDNA. cDNA was synthesized from mRNA using oligo(dT)_20_ primers and the Super-Script^™^ III First-strand Synthesis System for RT-PCR (Invitrogen, Carlsbad, CA). The cDNA samples, primers specific for the target genes (*TLR3*, *IFNβ*, *IFNγ*, *Mx1*, *OAS*, *IL6*, *IL8*, and *IL18*), *β-actin*, and SYBR Premix Ex Taq2 (PR081A, Perfect Real Time; TaKaRa Bio, Inc., Otsu, Japan) were prepared according to the manufacturer’s instructions. The sequences of all the primer pairs used in the quantitative real-time PCR analysis were previously described [29]. The quantitative real-time PCR analysis was run on a Chromo4 Real-Time PCR System (Bio-Rad Laboratories, Hercules, CA, USA) with the following cycle parameters: 95°C for 30 sec, followed by 40 cycles of 95°C for 5 sec and 60°C for 30 sec. The differences in gene expression were calculated by the 2^–ΔΔ^Ct method and were expressed as the fold change in gene expression as previously described [30]. *β-Actin* was used as the endogenous control for normalization of target gene expression [29]. The mean expression ± standard deviation was expressed as fold change and was compared to the expression in uninfected chickens.

### Genetic analysis of chickens

#### Sequence analysis of Mx cDNA

Whole blood was stored at -80°C until RNA isolation. Total RNA was extracted from samples using the RNeasy kit (Qiagen) according to the manufacturer’s instructions. cDNA was synthesized from mRNA using oligo(dT)_20_ primers (Life Technologies, Carlsbad, CA, USA) and the SuperScript^™^ III First-strand Synthesis System for RT-PCR according to the manufacturer’s instructions. Using cDNA as the template, PCR primers were designed to amplify two overlapping fragments encompassing the 1,700-base pair *Mx* cDNA sequence. The PCR primer sequences are as follows: 5′-TCTCCTTGCTGTGTGACTCT-3′ and 5′-AAGGAACTCCAAAAGTACAC-3′ for the first fragment and 5′-GGCGCTGAAAATGGCTCAAG-3′ and 5′-AGTGCAGCTTTGACAAGGGT-3′ for the second fragment. The 20-μL reaction mixture included 2.5 μL of 10× PCR buffer, 0.625 U of Ex Taq polymerase (TaKaRa), 1.5 mM MgCL_2_, 0.5 μM each primer, 0.2 mM each dNTP, and 100–500 ng of cDNA. The reaction conditions consisted of an initial denaturation step at 94°C for 3 min, followed by 30 cycles of 94°C for 30 sec, 60°C for 30 sec, and 72°C for 1.5 min. The first and second PCR products were 1,311 bp and 1,485 bp long, respectively. Direct sequencing was performed using the ABI prism BigDye Terminator Cycle Sequencing Kit with AmpliTaq DNA polymerase (Applied Biosystems, Carlsbad, CA, USA). The primers used for sequencing were the same as those used for the PCR. The genotype frequencies of each polymorphism for all breeds of chicken were determined and the differences were analyzed to examine their role in sensitivity to HPAIV infection.

#### Genotyping of the MHC Class I Genes *BF1* and *BF2*

Genomic DNA was extracted from whole blood samples collected before infection using the DNeasy Blood and Tissue Kit (Qiagen) to genotype the MHC class I genes *BF1* and *BF2*. The *BF1* and *BF2* PCR products were separately amplified by long-range (LR) PCR as previously described [31]. The primers pairs used for PCR amplification were *BF1*-Forward, 5′-CCTATTCCCCCAACAGGTTACGCCC-3′ and *BF1*-Reverse, 5′-ACAAGGGACCACAAGAGCTGTGCC-3′ and *BF2*-Forward, 5′-TTCCATCGGGTGTCCTTCGCC-3′ and *BF1*-Reverse, 5′-CACTGATCCCAAAGGAAGCCCTGG-3′. The 10-μL PCR amplification mixture contained 100 ng of genomic DNA, 1 U of KOD-plus-DNA polymerase (Toyobo Biologics, Otsu, Japan), 1× PCR buffer, 1 mM MgSO_4_, 200 μM each dNTP, and 0.5 μM each primer. The cycle parameters were an initial denaturation step at 98°C for 1 min, followed by 30 cycles of 98°C for 10 sec and 68°C for 3 min. The LR-PCR sizes of the *BF1* and *BF2* amplicons were 4,015 and 5,339 bp, respectively. The PCR products were directly sequenced using an ABI sequencing system (Applied Biosystems) according to the protocol for the Big Dye terminator method. The sequencing primers used in this study were designed against regions in exons 2 and 3. Assembly and database analyses were manually performed using Sequencher software (GeneCodes, Ann Arbor, MI, USA). The *BF1* and *BF2* alleles were tentatively named according to a previous study [32]. Briefly, the alleles were named by indicating the locus (*BF1* and *BF2*) and the chicken in which the sequences were observed (Thai indigenous chicken [TIC] or White Leghorn [WL]) using arbitrary numbering.

### Statistics

Kaplan-Meier survival curves for each group of chickens were constructed using the survival rate and infection period. Differences in the Kaplan-Meier survival curves were analyzed by the log-rank test using Bonferroni correction. Breed-wise differences in the macroscopic findings at death were analyzed by the Chi-square test. Differences in the virus titers of swab and tissue samples and cytokine expression were analyzed by the Mann-Whitney-U test. To compare the genotype frequency of SNPs in *Mx*, *BF1*, and *BF2* among the different breeds, Fisher’s exact test was employed. To evaluate the association between sensitivity to HPAIV and SNPs in *Mx*, *BF1*, and *BF2*, the chickens were divided into two groups based on time between inoculation and death: early phase (<48 h) and late phase (>49 h). Odds ratios and 95% confidence intervals were calculated by logistic regression analysis.

## Results

### Characteristics of the five H5N1 HPAI viruses

The nucleotide sequences of the protein-coding regions in all eight gene segments of four viruses, openbill-1604, crane-05, openbill-6-04, and pheasant-04, were determined (Accession numbers: EPI568201–EPI568208, EPI568209–EPI56821, EPI568217–EPI568223, and EPI568225–EPI568232, respectively). The nucleotide sequences of these four viruses, and that of pigeon-04, which was previously determined (AB576199-AB576206) [26], were compared. These viruses shared 99.1–100% and 98.6–100% homology and identity in the nucleotide and amino acid sequences, respectively. Among the 12 proteins predicted in these five viruses, there are 58 amino acid differences in 11 proteins (S1 Table). There are no differences in PA-X. Phylogenetic analysis of the *HA* gene showed that these five viruses belong to clade 1 of the WHO classification system (WHO/OIE/FAO H5N1 Evolution Working Group 2007). These viruses displayed high infectivity in embryonated eggs and MDCK cells, with titers ranging from 8.2–9.1 log_10_ EID_50_/mL.

### Lethality and clinical features of chicken inoculated with the five H5N1 viruses

**Fig 1:**
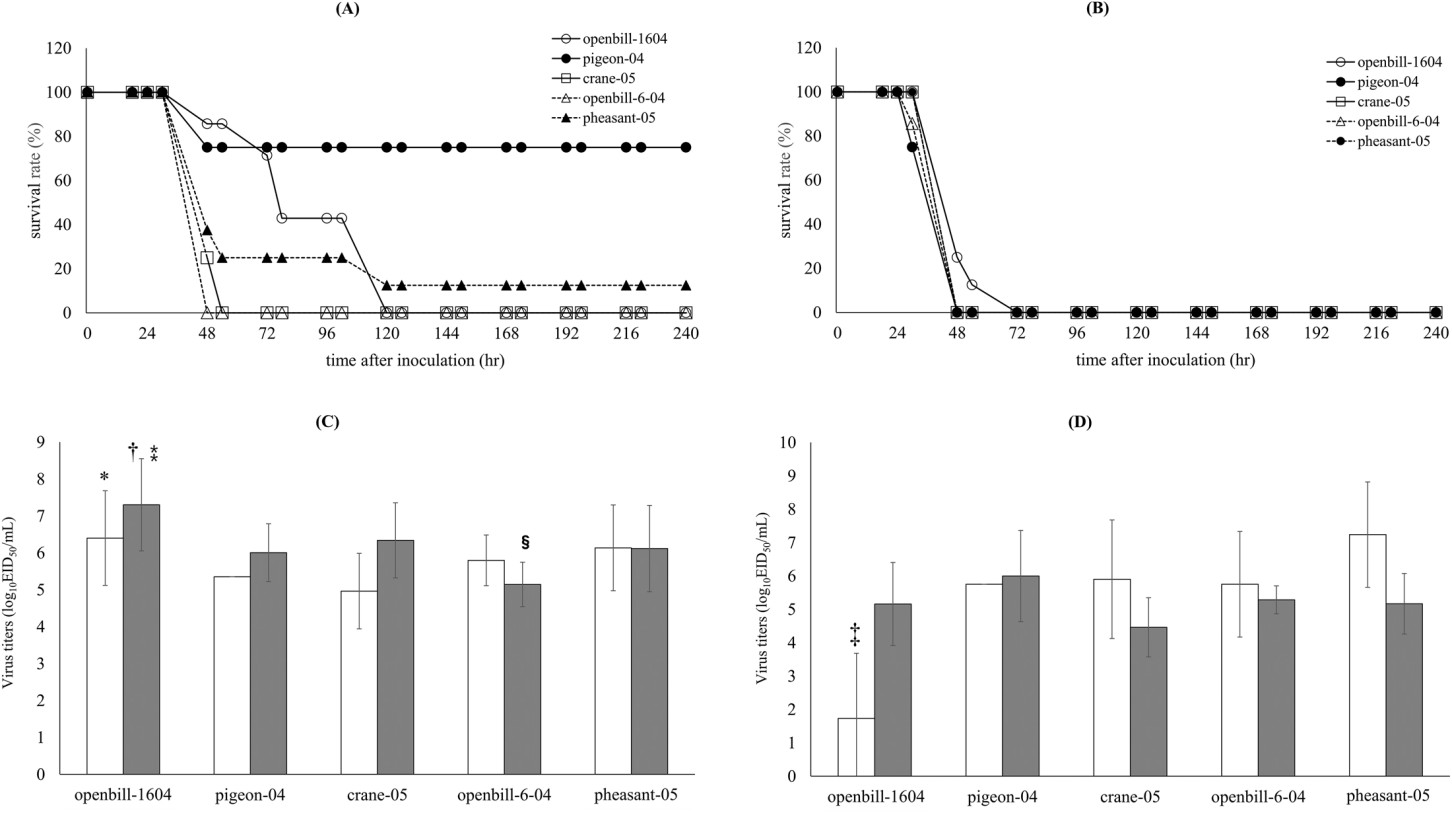
Lethality and viral shedding of five H5N1 HPAIVs. Survival curves of White Leghorns infected with five H5N1 HPAIVs at dosages of (A) 10^4^ EID_50_ and (B) 10^6^ EID_50_. Virus titers of trachea swabs (C) and cloacal swabs (D) obtained from dead White Leghorns infected with five HPAIVs at a dose of 10^4^ EID_50_ (white columns) and 10^6^ EID_50_ (gray columns). These swab samples were collected from the dead chickens immediately on the day of death. Virus titers are presented as the mean values ± standard deviation. * *P* < 0.05 vs. crane-05 (at 10^4^ EID_50_). † *P* < 0.05 vs. pigeon-04 at a dose of 10^6^ EID_50_. ⁑ *P* < 0.01 vs. openbill-6-04 at a dose of 10^6^ EID_50_. § *P* < 0.05 vs. pigeon-04 and crane-05 at a dose of 10^6^ EID_50_. ‡ *P* < 0.05 vs. crane-05, openbill-6-04, and pheasnt-05 at a dose of 10^4^ EID_50_.

The virulence of the five HPAIVs in chicken was compared by inoculating each virus at doses of 10^4^ EID_50_ and 10^6^ EID_50_ (Fig 1A and 1B). All chickens inoculated with 10^6^ EID_50_ of virus died within 48 h. Chickens inoculated with 10^4^ EID_50_ of virus displayed differences in the time between inoculation and death. All the chickens inoculated with openbill-1604, crane-05, pheasant-04, and openbill-6-04 died, whereas only two chickens inoculated with pigeon-04 died. Mean death time (MDT) and the clinical findings in each group of chickens are summarized in Tables 1 and 2. The survival rates differed significantly among the chickens inoculated with the different viruses at the lower dose (*P* < 0.01). Chickens infected with openbill-1604 at 10^4^ EID_50_ showed various macroscopic findings, such as swelling in the leg or face and discoloration of the comb or leg, at death. The MDT was also longer compared to that in other groups (*P* < 0.01).

**Table 1:** Summary of the experimental infections at 10^4^EID_50_.

Parameter	control	openbill-1604	pigeon-04	crane-05	openbill-6-04	pheasant-05
Number of infected animals	8	7	8	8	8	8
Number of dead chickens (%)	0	7 (100)	2 (25)	8 (100)	8 (100)	7 (87.5)
Mean death time (h)	-	78**†	48	48	48	48
(Range)	-	(48–120)	(48)	(48–72)	(48)	(48–120)
Macroscopic findings at death						
Swelling of leg	-	1/7	0/2	0/8	0/8	0/7
Swelling of face	-	4/7	0/2	3/8	5/8	0/7
Discoloration of comb	-	7/7§	0/2	0/8	0/8	0/7
Discoloration of leg	-	6/7§	0/2	0/8	0/8	0/7

**Mean death time of chickens inoculated with openbill-1604 was significantly longer than that of chickens inoculated with crane-05 or openbill-6-04 (P < 0.01).

^†^Mean death time of chickens inoculated with openbill-1604 was significantly longer than that of chickens inoculated with pheasant-05 (P < 0.05).

^§^Discoloration of the comb and leg occurred at a significantly higher rate in chickens inoculated with openbill-1604 than in chickens inoculated with crane-05, openbill-6-04, or pheasant-05 (P < 0.01).

**Table 2:** Summary of the experimental infections at 10^6^EID_50_.

Parameter	control	openbill-1604	pigeon-04	crane-05	openbill-6-04	pheasant-05
Number of infected animals	8	8	8	7	7	8
Number of dead chickens (%)	0	8 (100)	8 (100)	7 (100)	7 (100)	8 (100)
Mean death time (h)	-	48	48	48	48	48
(Range)	-	(48–72)	(30–48)	(48)	(30–48)	(48)
Macroscopic findings at death						
Swelling of leg	-	0/8	0/8	0/7	0/7	0/8
Swelling of face	-	2/8	8/8	4/7	0/7	0/8
Discoloration of comb	-	0/8	0/8	0/7	0/7	0/8
Discoloration of leg	-	3/8	0/8	0/7	0/7	0/8

### Viral shedding in the trachea and cloaca of dead chickens

Viral titers in tracheal and cloacal swabs collected from dead chickens were determined. Chickens inoculated with openbill-1604 at both 10^4^ EID_50_ and 10^6^ EID_50_ shed virus into the trachea at a significantly higher titer than chickens inoculated with other viruses (Fig 1C). However, viral shedding from the cloacal swabs of chickens inoculated with 10^4^ EID_50_ virus was significantly lower than the shedding from chicken infected with the higher inoculum (*P* < 0.01; Fig 1D). Virus was not recovered from the five surviving chickens in the pigeon-04 group.

### Distribution of virus in chickens 24 h after inoculation

**Fig 2:**
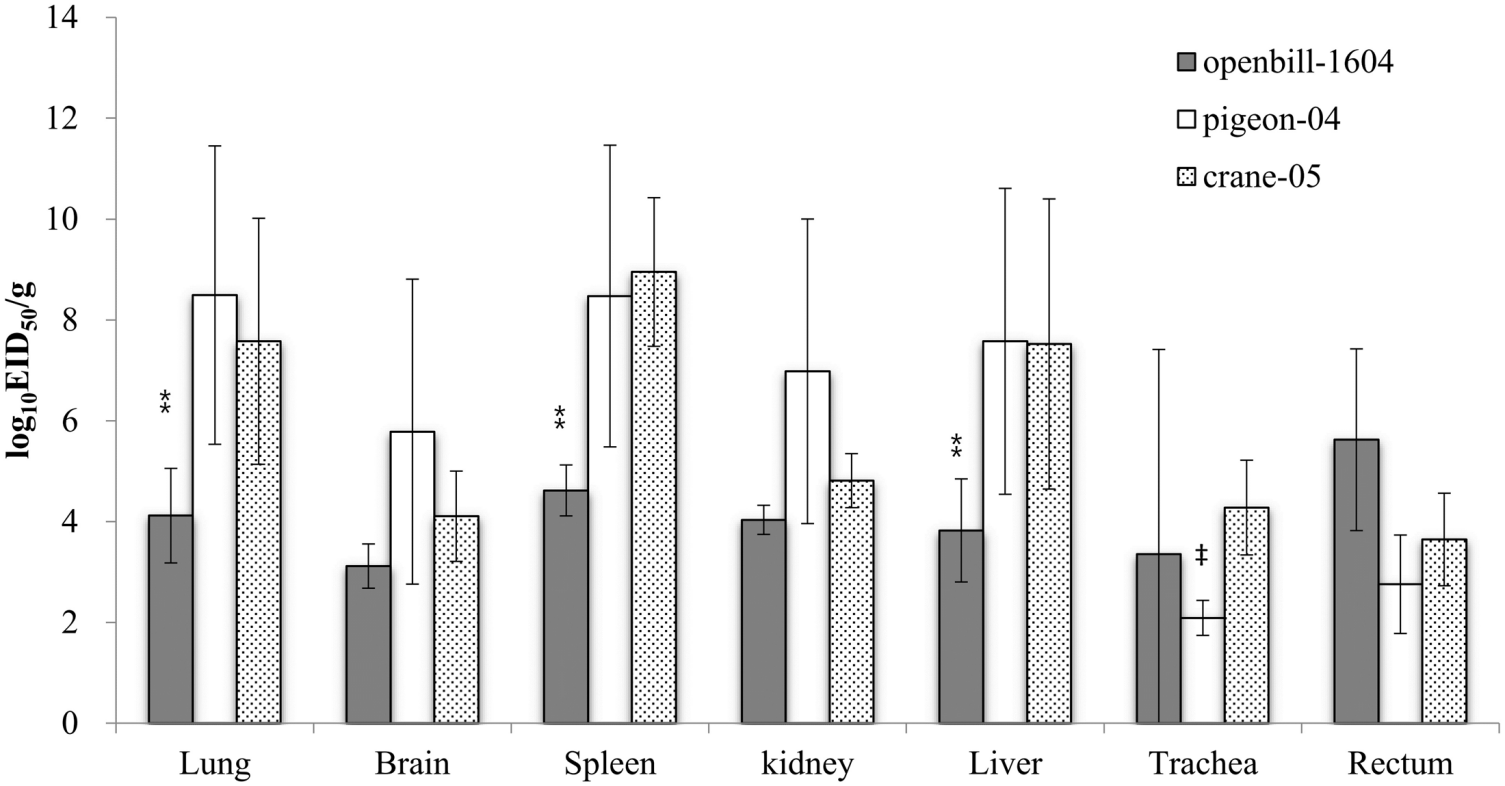
Viral distribution in chickens infected with three HPAIVs at a dose of 10^6^ EID_50_. Three chickens in each group were sacrificed at 24 h post-inoculation. Virus titers are presented as the mean values ± standard deviation. ⁑*P* < 0.01 vs. pigeon-04 and crane-05. ‡ *P* < 0.01 vs. crane-05.

To compare viral distribution and replication in host organs, three HPAIVs (openbill-1604, pigeon-04, and crane-05) were inoculated into three chickens. The viral titers in the lungs, spleen, and liver of chickens inoculated with openbill-1604 were significantly lower than those in the organs of chickens inoculated with pigeon-04 and crane-05 at 10^6^ EID_50_ (*P* < 0.01; Fig 2). In the pigeon-04-infected group, the viral titers in the trachea were significantly lower than those in the crane-05-infected group. No virus was recovered from the examined organs of chickens infected with HPAIV at 10^4^ EID_50_.

### Host response in chickens infected with three strains of HPAIV

In all three HPAIV-infected groups, at both 10^4^ and 10^6^ EID_50_, the expression of *IFN-β*, *IFN-γ*, *TLR3*, and *IL18* in the lung was low. However, the expression of *IL-6* and two interferon-stimulated genes, *OALS* and *Mx1*, were upregulated in the 10^6^ EID_50_-inoculated groups. In the brain and spleen, cytokine mRNA levels were upregulated.

**Fig 3:**
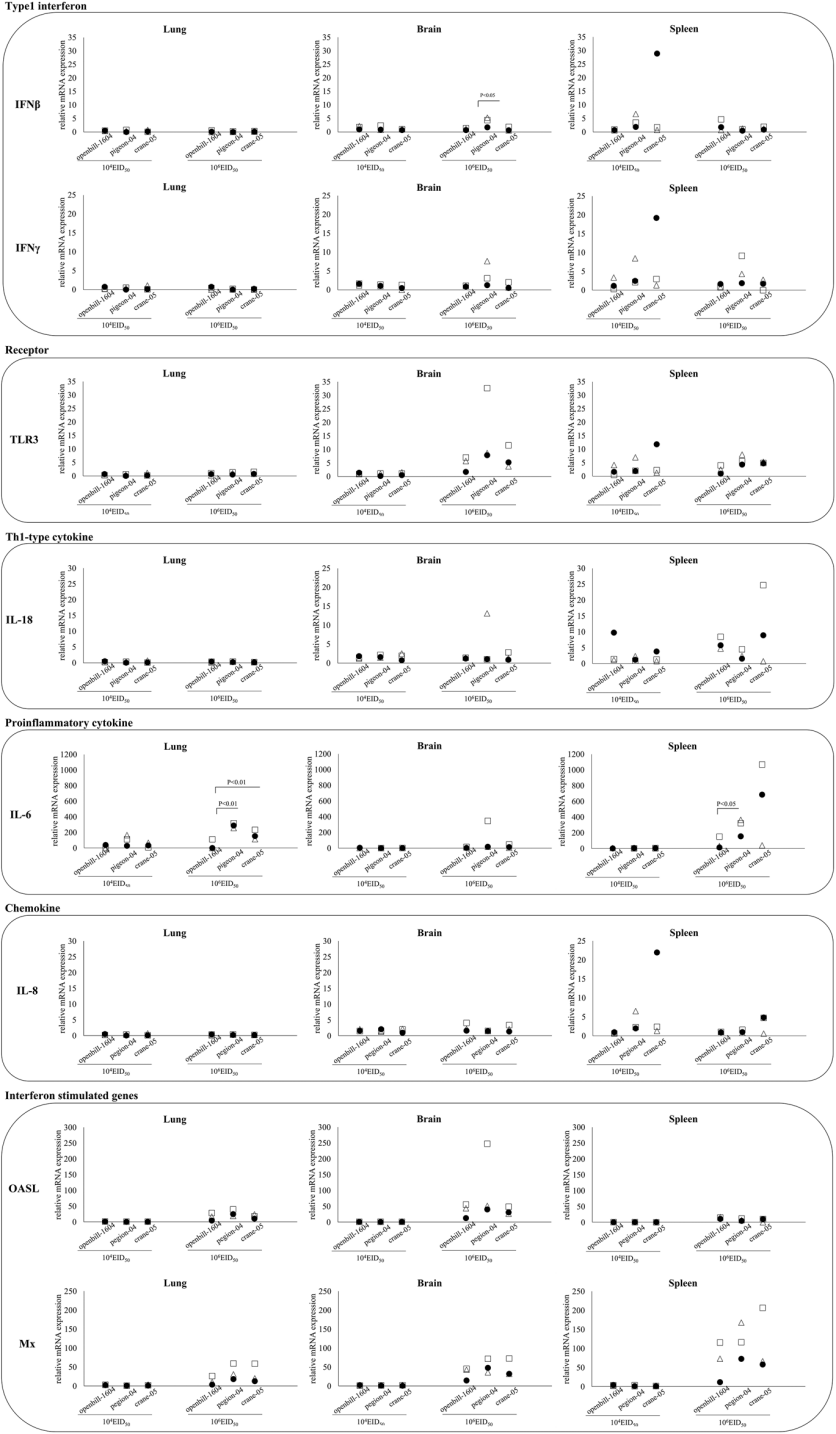
Comparison of the host gene responses in the lung, brain, and spleen in chickens infected with three HPAIVs. The level of mRNA expression of each gene was examined by real-time PCR analysis using specific to the corresponding gene. mRNA leveles indicate mean values ± standard deviations.

A comparative analysis of the chickens infected with different viruses showed that the expression of *IL-6* in the lungs of openbill-1604-infected chickens was significantly lower than that in pigeon-04- and crane-05-infected chickens (*P* < 0.01), as well as in the spleen of pigeon-04-infected chickens (*P* < 0.05). *IFNβ* expression in the brain was higher in pigeon-04-infected chickens than in openbill-1604- and crane-04-infected chickens at 10^6^ EID_50_ (Fig 3).

### Lethality and viral shedding in four breeds of Thai indigenous chicken inoculated with pigeon-04

**Fig 4:**
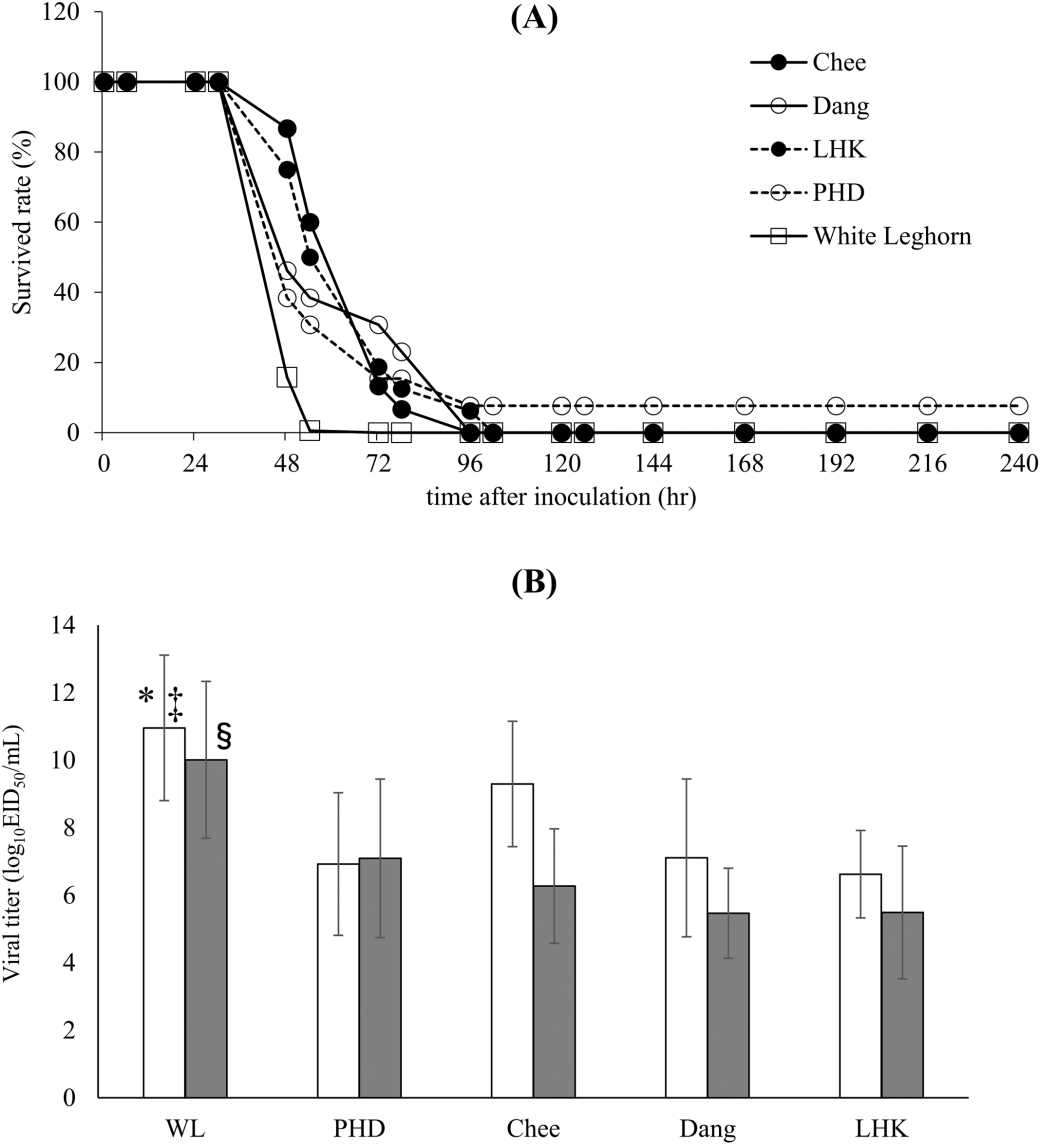
Lethality and viral shedding of pigion-04 in three breeds of Thai indigenous chicken and White Leghorn chicken. (A) Survival rate of chickens after intranasal inoculation with pigeon-04 at a dose of 10^5^ EID_50_. (B) Virus titers of tracheal (white columns) and cloacal (gray columns) swabs from dead chickens. * *P* < 0.05 vs. Chee. ‡ *P* < 0.01 vs. PHD, Dang, and LHK. § *P*<0.01 vs. PHD, Chee, Dang, and LHK.

As pigeon-4 displayed lower pathogenicity in White leghorn chickens at 10^4^ EID_50_, it was inoculated at 10^5^ EID_50_ into five breeds of chicken (PHD, Chee, Dang, LHK, and White Leghorn) to determine if there are any differences in susceptibility to HPAIV among these breeds. All chickens, except one PHD chicken, died because of virus inoculation. One PHD chicken survived for 10 days after inoculation without displaying any clinical symptoms, and this chicken did not seroconvert at 10 days after inoculation. The MDT was extended in three Thai indigenous breeds, Chee, Dang, and LHK, compared to that of White Leghorn; the MDT of White Leghorn was 48 h (range, 48–72 h), whereas the MDT of Chee, Dang, and LHK were 72 h (range, 48–96 h), 54 h (range, 48–96 h), and 63 h (range, 48–102 h), respectively. The Mann-Whitney test showed that the MDT of White Leghorn was significantly shorter than those of Chee (*P* < 0.01), LHK (*P* < 0.01), and Dang (*P* < 0.05). The MDT of PHD was 48 h (range, 48–96 h), which was not different from that of White Leghorn. No significant differences were observed among the different breeds of Thai indigenous chicken (Fig 4A). Analysis of the differences in the Kaplan-Meier survival curves between the various breeds of chicken by log-rank test showed significant differences between White Leghorn and Chee (*P* < 0.01), Dang (*P* < 0.01), and LHK (*P* < 0.05). No significant differences were observed among the Thai indigenous chicken.

The postmortem swab titer in the trachea of White Leghorns was significantly higher than that in the trachea of Chee (*P* < 0.05), PHD, Dang, and LHK (*P* < 0.01 each; Fig 4B). The swab titer in the cloaca of White Leghorns was also significantly higher than that in all four breeds of Thai indigenous chickens (*P* < 0.01). There were no significant differences observed among the Thai indigenous chicken breeds.

### *Mx* cDNA analysis

*Mx* cDNA was analyzed in all infected chickens. The complete sequence (2,032 bp) of the *Mx* cDNA was determined in the Thai indigenous chicken breeds (GenBank accession numbers: LC033614–LC033670) and White Leghorn (GenBank accession numbers: LC033671– LC033685), and 14 nucleotide polymorphisms with accompanying amino acid substitutions were identified (S2 Table). When the allele frequencies were compared among the various breeds, significant differences were observed for the substitutions at positions 5, 31, 41, 42, 171, 199, 308, 339, 552, and 631. Substitutions at sites 41 and 42 were identified in the same chicken. The odds ratio and 95% confidence interval for each substitution are shown in S2 Table. No significant association was observed between the substitutions in the *Mx* gene and sensitivity to HPAIV.

### Polymorphism analysis of *BF* genes

Twenty alleles in *BF1* loci (BF1*TIC1-*TIC20; GenBank accession numbers: LC033686–LC033754) and 19 alleles in *BF2* (GenBank accession numbers: LC033759–LC033777) were detected in Thai indigenous chickens, and 14 *BF1* and 14 *BF2* alleles were newly identified (S3 Table). Unfortunately, some of the *BF1* and *BF2* alleles from 12 chickens were not assigned by Sanger sequencing due to the observation of ambiguous nucleotides. Four alleles in *BF1* loci (BF1*WL1-*WL4; GenBank accession numbers: LC033755–LC033758) and seven alleles in *BF2* loci (BF2*WL1-*WL7; GenBank accession numbers: LC033778-LC033783) were identified in White Leghorns, of which, one *BF1* allele and two *BF2* alleles were novel.

The allele frequency ranged from 3.3% to 38.5% in *BF1* and *BF2* (S3 Table). Among the different breeds, the distribution of both alleles was significantly different (*P < 0*.*01*). Comparison of the allele frequencies of *BF1* and *BF2* to sensitivity to HPAIV infection revealed no significant association.

## Discussion

Although the H5N1 HPAIV strains used in this study were genetically closely related and show 100% lethality in White Leghorn chickens at 10^6^ EID_50_, there were obvious differences in pathogenicity when the chickens were inoculated with 10^4^ EID_50_ of virus. Crane-05, openbill-6-04, and openbill-1640 showed 100% lethality at 10^4^EID_50_; however, the MDT of openbill-1640 was extended compared to that of the other viruses, and the infected chickens displayed variable clinical symptoms. Viral shedding in the trachea of dead chickens was significantly higher in openbill-1640 than in other H5N1 isolates (Fig 1C), while that in cloaca was significantly lower (Fig 1D). In HPAIVs, the virus shedding patterns have been reported to be varied in the different isolates [33]. Because viral titers in organs of chicken infected with openbill-1604 at 24 h after inoculation were also lower than other viruses (Fig 2), this might result in the lower titer shedding from cloaca.

Among the viruses examined, pigeon-04 showed the mildest pathogenicity in White Leghorn, with a 75% survival rate at 10^4^ EID_50_, and conversely, openbill-6-04 showed the 100% mortality with shorter time to death (Fig 1A). These observed differences in virus pathogenicity could be due to slight differences in the viral genomes. Pigeon-04 possess several unique amino acid substitutions, at positions 451 of PB2, 106 of PB1, 74 and 75 of PB1-F2, 584 of PA, and 492 of NP. Openbill-6-04 possessed dual amino acid substitutions at positions 627 and 701 in the PB2 genes, which affects polymerase activity in chickens and mice [6,7]. This strain also possessed the other many unique substitutions at 643, 644 and 752 of PB1, 11 and 192 of PA, 59, 152, 231, 451 and 518 of HA, 111, 146 and 266 of NA, 86 of M2, 76, 147 and 166 of NS1, and 14 of NS2. Among these substitutions, the nucleotide sequences at 192 codon of PA were UGU in openbill-6-04, and CGU in the other isolates. CGU is a rare codon that stimulates the ribosomal +1 frameshifting, resulting in the PA-X expression [34,35]. Substitution from CGU to UGU that exhibits higher codon usage frequency may affect PA-X expression, resulting in alteration of virulence governed by this protein [34–37], although relationship between frameshift efficay and codon usage remained to be elucidated [35]. The one or a combination of these other amino acid differences may affect viral replication and pathogenicity in chickens. Further evaluation of these substitutions using reverse genetics techniques to construct individual mutants is required to determine the contribution of each substitution to pathogenicity.

Type 1 interferon exerts an antiviral effect through activation of innate immune cells such as macrophages and NK cells. A previous study showed that A/Vietnam/1203/2003 (H5N1) induced strong inflammatory and Th1-associated cytokines in multiple organs of chickens [38]. Another study showed that a highly virulent H5N1 virus induced antiviral (*IFN-α* and *IFN-β*) and proinflammatory (*IL-4*, *IL-6*, *IL-8*, and *IL-15*) cytokine mRNA expression in the lung at 24 h post infection, which abruptly decreased within the next 8 h [39]. Kuchipudi et al., also reported the highly elevated immune and proinflammatory response following H5N1 HPAIV infection in chicken cells, and that the response is mediated in part by the inhibition of signal transducer and activator of transcription-3 (STAT-3) [40]. The enhanced pathogenicity of H5N1 HPAIV in chickens may be associated with the extremely rapid replication of the virus in macrophages and vascular endothelial cells, which disrupts the innate immune response [39,40]. In our study of chickens inoculated with three viruses, interferon expression in the lungs was not upregulated at 24 h after inoculation, even though the interferon-stimulated genes *OASL* and *Mx* (Fig 2) were upregulated. This suggests that peak interferon expression might have occurred at an earlier time point. In contrast, at 24 h post inoculation, the expression of these cytokines was slightly elevated in the brain and spleen. Although the changes in each tissue were not evaluated as a time course, we hypothesize that the timing of cytokine upregulation in the lung, brain, and spleen may differ. A comparison of the cytokine responses among chickens inoculated with the three different viruses showed that IL-6 expression in the lungs and spleen was significantly lower in chickens infected with openbill-1604 than in chickens infected with the other viruses. As openbill-1604-infected chickens showed lower viral loads, the difference observed in cytokine levels might be associated with the differences in viral multiplication. On the other hand, although pigeon-04 showed the least mortality at a dose of 10^4^EID_50_, virus multiplication and IL-6 expression in organs were similar to crane-05 that showed the highest mortality (Fig 3). Some other host factors as well as viral genomic factors described above, might affect the differences. For understanding why there was a pathogenicity difference where virus titers were not appreciably different, a global gene expression analysis, such as microarray or RNA sequences, may reveal such host factors that are differentially regulated in chicken infected with the different strains.

The commercial breed of chicken used in this study, White Leghorn, appeared to be more sensitive to HPAIV than the Thai indigenous breeds. Differences in sensitivity among breeds of chickens have been reported for other diseases, such as Marek’s disease [41], and Sironi et al. reported variable responses of different chicken lines to HPAIV infection [15]. In addition, Kalaya et al. reported that some Thai indigenous chickens displayed resistant traits during the H5N1 outbreak in 2003–2004 [16]. In our study, we demonstrated that White Leghorns appeared to be more sensitive to pigion-04 infection than the Chee, Dang, and LHK lines of Thai indigenous chickens, as evaluated by the MDT and virus shedding. Based on the results of a microsatellite analysis, Thai indigenous chickens are believed to be closely related to original domesticated chickens [42]. Due to the genetic diversity displayed by the indigenous chicken population, they are expected to present unique genotypes and traits. Mx proteins confer resistance to different viral families, and the antiviral activity of *Mx* gene products has been described in several vertebrate species [43,44]. *Mx* gene expression is induced by interferon [45]. The *Mx* gene product has been suggested to be a major component of resistance to influenza virus in mice [46,47], and a serine to asparagine substitution at position 631 is thought to confer antiviral activity [19]. However, in some *in vivo* studies, no association has been found between the *Mx* genotype at position 631 and the response of chickens towards H5N1 and H7N1 HPAIV infection [15,48]. In a previous *in vitro* study, asparagine 631 allele of chicken Mx did not inhibit influenza virus replication [22]. Likewise in the present study, no statistical association was found between the *Mx* genotype at residue 631 and the survival time after inoculation with HPAIV. Although no association with resistance to HPAIV infection was found, we identified 13 other polymorphic residues in the *Mx* gene. Four polymorphisms (Mx31, 94, 171, and 552) were newly discovered in this study, and nine others were previously found in different Japanese and Egyptian indigenous chickens [19]. Among these polymorphisms, 10 displayed significantly different frequencies among the chicken breeds (S2 Table).

Some MHC alleles have been shown to confer resistance against virus infection, and in this study, we identified 24 and 26 alleles of *BF1* and *BF2*, respectively, in Thai indigenous chicken lines and White Leghorns. Of these, 15 putative *BF1* loci, 16 *BF2* loci, and the α1 and α2-coding sequences appear to be novel. The chickens used in this study were native breeds, and high variability in the *BF* loci and novel alleles were observed. Previous studies have reported that the MHC-B21 haplotype was associated with resistance to H5N1 virus infection, with a 100% survival rate. In contrast, chicken with the MHC-B13 haplotype showed 100% mortality during HPAI outbreaks in Thailand [16]. The MHC-B21 haplotype is also associated with lower tumor-related mortality due to Marek’s herpes virus infection than other haplotypes [49]. In this study, BF2*WL1 and BF1*WL1 showed 100% homology to the MHC-BF21 haplotype reported in a previous study [41]. However, there did not appear to be an association between these alleles and sensitivity to HPAI infection in our study. Hunt et al. performed an experiment using a series of MHC congenic White Leghorn chicken lines (B2, B12, B13, B19, and B21), and reported that none of the lines were completely resistant to a lethal challenge, as evidenced by mortality rates ranging from 40–100% [50]. In our study, although the Thai indigenous chickens had variable BF alleles, whether a specific BF allele was related to resistance against HPAIV could not be determined. Non-MHC immune-related genes, for example dsRNA dependent protein kinase (PKR) [51,52], 2’, 5’-Oligoadenylate synthetase (OAS) [53], and Smad7 [54], and/or a combination of such genes with particular MHC alleles may contribute to the sensitivity of chickens to HPAIV infection. More global genetic analysis on immune related genes, using next generation sequencing technologies would be help to the elucidation.

In conclusion, here we demonstrated that genetically similar H5N1 HPAIVs showed different pathogenicity, which may be dependent on differences in the efficiency of viral replication or tropism. Because of the constraints regarding laboratory setting in the BSL-3 facility, we were not able to perform kinetic analysis of the viral replication in inoculated chickens, such analysis would deepen our understanding on the viral replication of those isolates in vivo. Also, scrutiny on transmission ability of those isolates in chickens would have provided further insights in pathogenicity of those isolates, as one of the isolates, pheasant-04, showed longer survival time than the crane-05 while there were no appreciable differences in the virus shedding in the cloaca. Beyond such constrains, we demonstrated that one strain of H5N1 HPAIV, pigeon-04, induced an extended MDT in Thai indigenous chickens. Although we could not identify the host factor associated with increased resistance to HPAIV, the genetic diversity of Thai indigenous chickens may be useful tool for determining the host defense mechanism against HPAIV through extensive genetic analyses.

## Supporting Information

S1 Table Amino acids differences between five different HPAIV. (TIF)

S2 Table Allele frequency (%) in *Mx1* among four breeds of Thai indigenous chickens and White Leghorns. (TIF)

S3 Table Summary of *BF1* and *BF2* alleles and their frequency in Thai indigenous chickens and White Leghorn. (TIF)
